# Effect of nickel-coated carbon nanotubes on the preparation and wear resistance of microarc oxidation ceramic coating on ZL109 aluminum alloy

**DOI:** 10.1038/s41598-022-15210-w

**Published:** 2022-06-30

**Authors:** Jiang Liu, Xinhe Zhu, Dengqing Ma, Jingguo Fu, Wenbin Xue, Fan Zhang, Chunsheng Ma

**Affiliations:** 1 College of Marine Engineering, Dalian Maritime University, Dalian, 116026 China; 2 Shenzhen Academy, Dalian Maritime University, Shenzhen, 518063 China; 3 College of Nuclear Science and Technology, Beijing Normal University, Beijing, 100875 China

**Keywords:** Ceramics, Mechanical properties, Metals and alloys

## Abstract

In order to adapt to the development of lightweight equipment, and further improve the wear resistance of ZL109 aluminum alloy, the influence of nickel-coated carbon nanotubes as an electrolyte additive on the preparation and wear resistance of microarc oxidation ceramic coatings on ZL109 aluminum alloy surface was investigated. In this work, 0.4 g/L, 0.8 g/L, 1.2 g/L, 1.6 g/L, and 2 g/L nickel-coated carbon nanotubes were added to the electrolyte respectively. The microarc oxidation ceramic coatings were prepared under bipolar pulse constant pressure mode, which were analyzed from the aspects of morphology, chemical composition, and wear resistance property. The results show that the nickel-coated carbon nanotubes possess a great influence on ceramic coatings. The morphology of ceramic coatings was significantly changed. In this work, the coating prepared by 1.2 g/L nickel-coated carbon nanotubes exhibits excellent wear resistance property.

## Introduction

In pursuit of lightweighting, a trend of employing aluminum alloys in the field of key components of equipment is formed^1^. ZL109 aluminum alloy has an excellent casting and airtight performance, low linear expansion coefficient and density, is widely used in the internal combustion engine piston and other parts^2^. However, the wear resistance of cast aluminum alloy is limited^3–5^. Furthermore, the development of high reinforcement technologies poses a more severe challenge to the wear resistance of aluminum alloy parts. Therefore, surface treatment technologies such as magnetron sputtering^6^, surface spraying^7^, electroplating^8^, microarc oxidation^9^, anodic oxidation^10^, and laser surface cladding^11^ are widely used on the aluminum alloy surface. However, there are some deficiencies in the application prospect, service life, manufacturing cost, and capacity of adapting to complex load for the existing technologies^12–15^.

Microarc oxidation (MAO) is a promising technology in the area of surface strengthening for light alloys^16–19^. In order to further improve the performance of MAO ceramic coatings, researchers have carried out a lot of research on MAO electrical and electrolyte parameters, and achieved some remarkable results^20–23^. Carbon nanotubes are a kind of one-dimensional quantum material with excellent mechanical properties, heat transfer properties, electrical conductivity, and flexibility. Therefore, they are widely used as matrix strengthening additives^24–27^. In the field of MAO, some scholars have studied the influence of carbon nanotubes on the preparation and properties of magnesium and titanium alloy MAO ceramic coatings^28,29^. However, the effect on the growth and properties of ZL109 aluminum alloy MAO ceramic coatings is rarely reported. Furthermore, in the process of microarc discharge, an electromagnetic field is formed in the electrolyte^30^. Therefore, the authors propose to add magnetic nanoparticles, namely nickel-coated carbon nanotubes (NCNs), into the electrolyte to investigate the effect of magnetic nanoparticles additives on the MAO reaction process and wear resistance of the coatings. This work has certain reference value for the development of MAO electrolyte additives which is significant for improving MAO coatings performance.

## Experimental details

**Figure 1:**
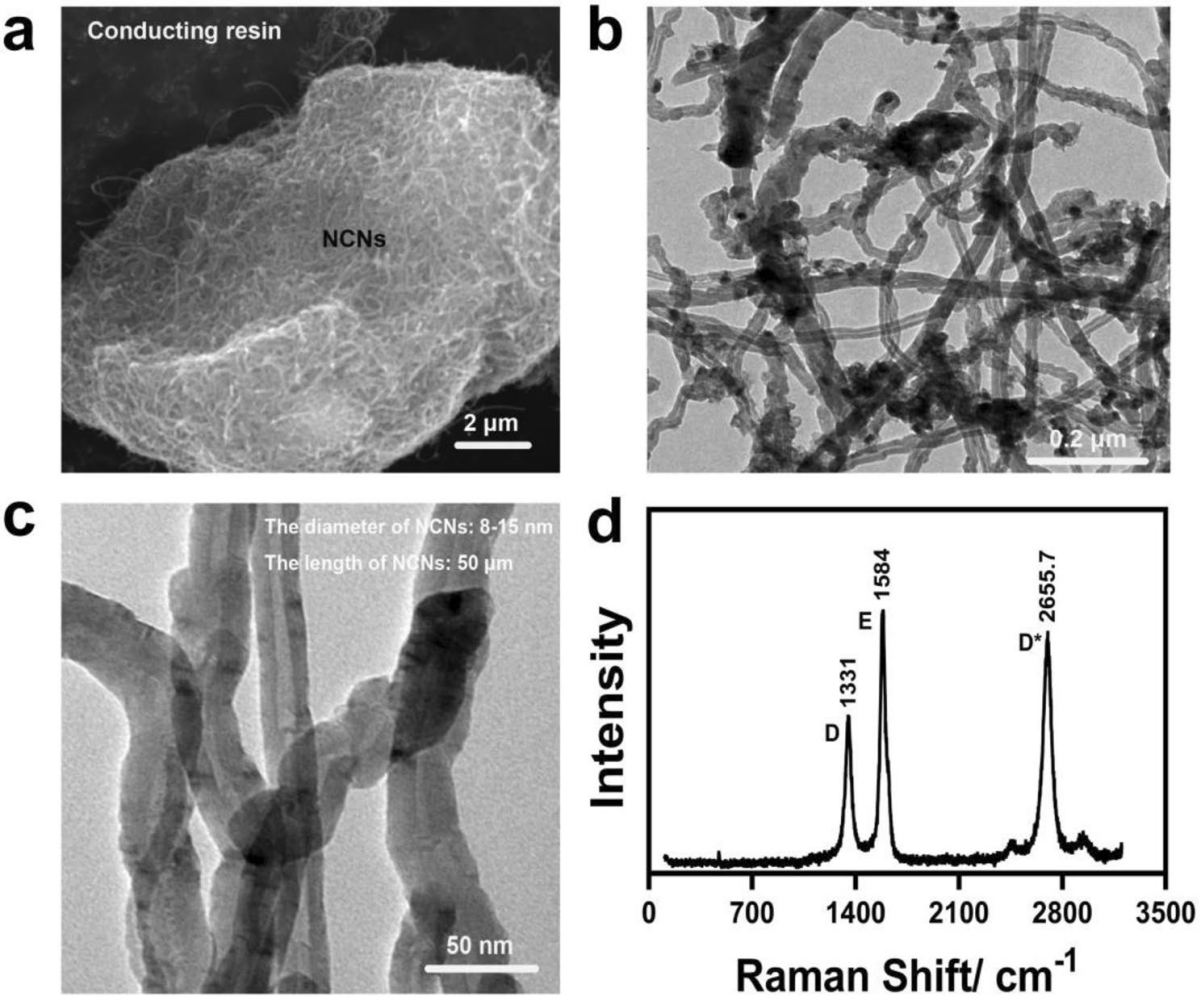
The morphology, size and Raman spectra of NCNs (OriginPro 2018, https://www.originlab.com/index.aspx?go=Products/Origin; Adobe Illustrator CS5, https://www.adobe.com/cn/products/illustrator.html#scroll).

The ZL109 aluminum alloy (wt%: 11–13% Si, 0.5–1.5% Cu, 0.8–1.3% Mg, 0.8–1.5 Ni, Residue Al) samples (40 mm * 10 mm * 10 mm) were used to fabricate MAO ceramic coatings by a self-developing power supply. The MAO electrolyte was composed of 4 g/L Na_2_SiO_3_,4 g/L Na_2_WO_3_,2 g/L KOH,2 g/L EDTA-2Na, NCNs (Beijing Deke Daojin Science And Technology Co., Ltd.), Polyethylene glycol (mass ratio to nickel-coated carbon nanotubes 5:1), and DI water. The size, morphology, and Raman spectra of NCNs are shown in Fig. 1. The Raman spectra (Fig. 1d) shows that the NCNs is a kind of multiwalled carbon nanotubes, and it has certain defects. As the content of Ni is low, there is no obvious peak for Ni in Raman spectra. The concentration of NCNs was set separately to 0 g/L, 0.4 g/L, 0.8 g/L, 1.2 g/L, 1.6 g/L, 2.0 g/L. The bath was stirred by a magnetic stirrer at a speed of 150 rpm for 60 min and then ultrasonic oscillations for 1 h by using an ultrasonic cleaner before the MAO. The positive voltage of MAO power supply was set in three stages. The total reaction time for each MAO treatment is 12 min. The detailed protocol for the MAO electrical parameters is shown in Table 1.

**Table 1:** The protocol of the MAO electrical parameters.

Stages	Positive voltage (V)	Negative voltage (V)	Frequency (Hz)	Duty cycle	Reaction time (min)
1	340	120	500	20%	4
2	380	120	500	20%	4
3	420	120	500	20%	4

A scanning electron microscope (SEM, VEGA 3, TESCAN) is employed for the thickness, surface, and cross-section morphologies of MAO coatings. X-Ray Photoelectron Spectroscopy (XPS) on phi5000VersaProbe and an energy dispersive spectrometer (EDS) incorporated in the SEM system are used for the surface analysis. The phase composition is analyzed by X-ray diffraction (XRD, EMPYREAN, Bragg–Brentano Geometry, 40 kV, 40 mA, Cu target, 5°/min). An Optical Profilometer (CONTOURGT) is employed for the surface roughness and three-dimensional shape of the coatings. The porosity and mean pore size of coatings are analyzed by the Image J software. The real-time voltage and current data are captured by an acquisition card that comes with the MAO power supply. The microhardness of coatings is detected by a micro vickers (HVS-1000Z). The detection of Raman spectra is carried out by an atomic force microscope-Raman spectroscopy (Time of exposure: 60 s, power: 1%, Integral number of times: 1). The sclerometric test is carried out by a coating adhesion automatic scratch instrument (WS-2005), the load is 50 N, the loading rate is 50 N/min, the scratch rate is 3 mm/min, the processing time is 2 min.

Reciprocating friction–wear tests are carried out to analyze the wear resistance of the MAO coatings. In the friction-wear tests, the sliding counterbody material is the boron copper cast iron (110 mm * 10 mm * 2 mm). The test load is 40 N; the sliding speed is 0.2 m/s; the test time is 30 min, and the sliding distance is 40 mm. The weight loss of samples is surveyed by an electrical balance (0.1 mg, JJ224BC). In order to minimize data fluctuations, four samples of each experimental condition were measured for each data, the reported results are the average values. The condition of lubricating was set to lean oil lubrication for simulating poor lubrication conditions.

## Results and discussion

### The morphology of MAO coatings

**Figure 2:**
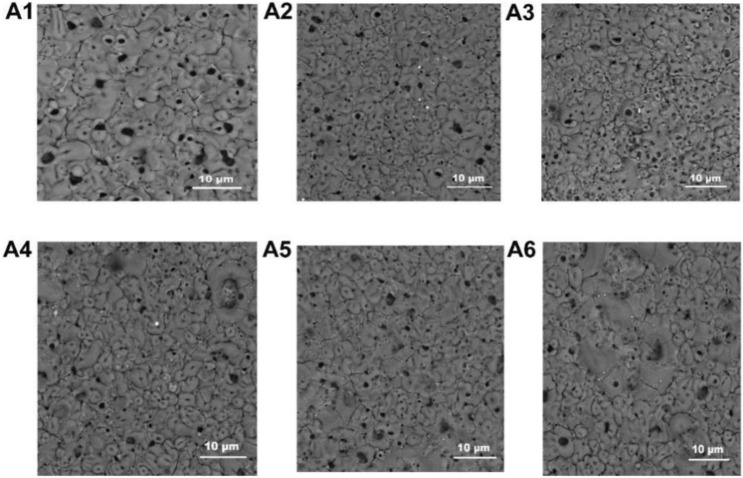
The surface morphologies of the MAO coatings (**A1**): The concentration of NCNs is 0 g/L; (**A2**): 0.4 g/L; (**A3**): 0.8 g/L; (**A4**): 1.2 g/L; (**A5**): 1.6 g/L; (**A6**): 2.0 g/L; Adobe Illustrator CS5, https://www.adobe.com/cn/products/illustrator.html#scroll).

**Figure 3:**
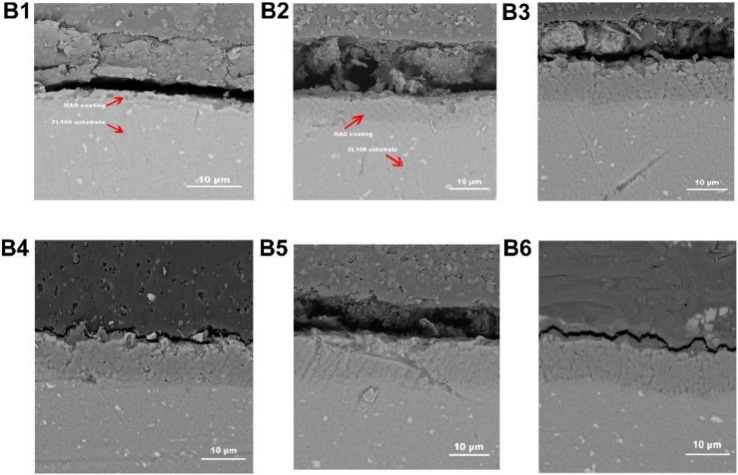
The cross-section morphologies of the MAO coatings (**B1**): The concentration of NCNs is 0 g/L; (**B2**): 0.4 g/L; (**B3**): 0.8 g/L; (**B4**): 1.2 g/L; (**B5**): 1.6 g/L; (**B6**): 2.0 g/L; Adobe Illustrator CS5, https://www.adobe.com/cn/products/illustrator.html#scroll).

The surface morphologies of MAO coatings are shown in Fig. 2. The cross-section morphologies of MAO coatings are given in Fig. 3.

The carbon nanotubes possess a unique and symmetrical electronic structure. Therefore, they have high storage dielectric micro-charging ability. According to the microarc oxidation gas film discharge theory, the electric field at both ends of the gas film is enhanced by the carbon nanotubes, and the breakdown voltage of the microarc oxidation reaction will be reduced. Then, the quality of MAO coatings is improved, and the coatings become denser. In addition, the good conductivity and high thermal conductivity are beneficial to surface current uniformity, which further improves the quality of MAO coatings. Meanwhile, there is a remarkable gradient magnetic field in the electrolyte during the MAO process. The nickel coating on carbon nanotubes results in the enrichment of carbon nanotubes on the surface of samples, which enhances the effect of carbon nanotubes.

**Figure 4:**
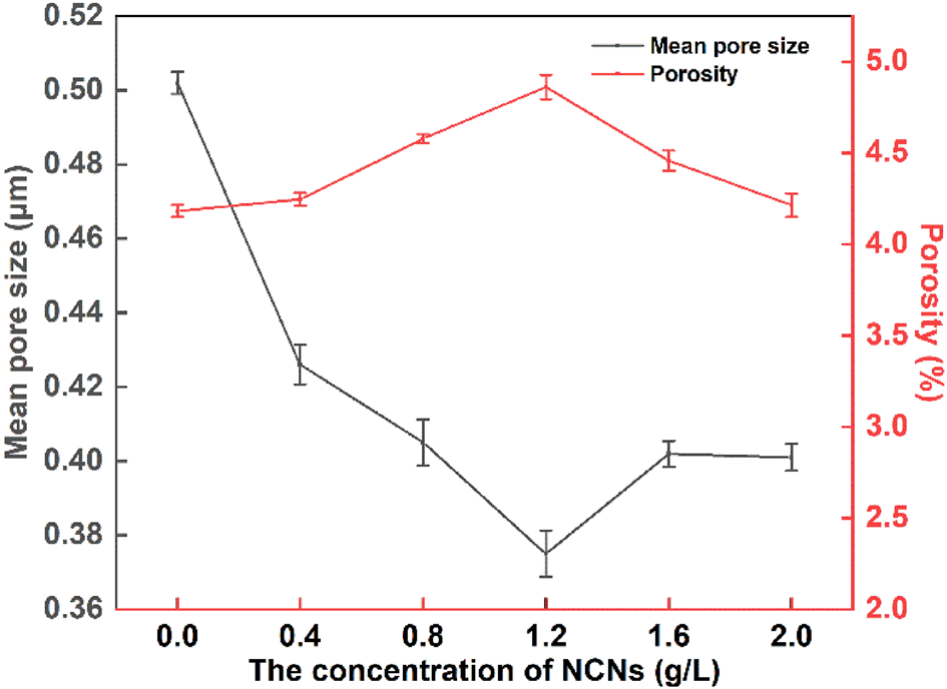
The surface porosity and average pore size of the MAO coatings (OriginPro 2018, https://www.originlab.com/index.aspx?go=Products/Origin).

The impact of NCNs on the preparation of MAO coatings can be distinctly found in Figs. 2 and 3. The surface of the MAO coating without NCNs additive shows obvious porous morphology, and pore size is larger than the others. The porosity and mean pore size of the MAO coatings affected by NCNs exhibit a fascinating trend with the introduction of NCNs (Fig. 4), and the crateriform protuberances appear with increasing the concentration of NCNs (Fig. 2A4,A5,A6).

**Figure 5:**
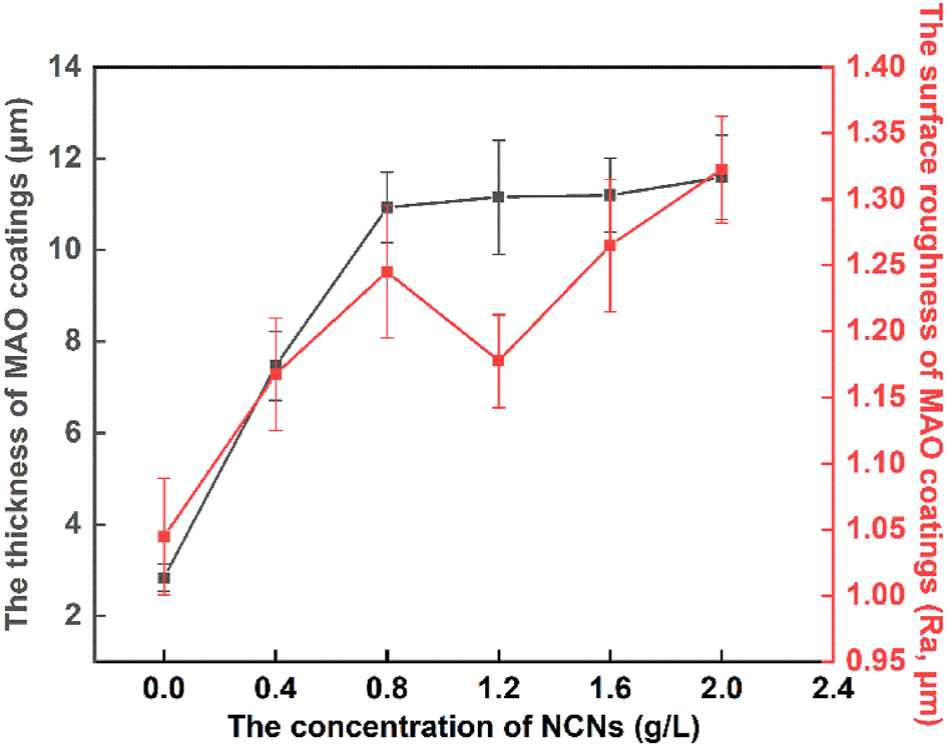
The thickness and surface roughness of the MAO coatings (OriginPro 2018, https://www.originlab.com/index.aspx?go=Products/Origin).

On account of the NCNs, the quality of the coatings is improved, then, the mean pore size is reduced dramatically; the distribution of surface current is more uniform, then, the micro-charge is well-distributed, and the porosity increases accordingly. Meanwhile, under the gradient magnetic field in the electrolyte, the NCNs gather on the surface of samples. When the concentration of NCNs is too high, the thickness of the MAO coatings reaches a higher level (Fig. 5), and the coatings become more compact (Fig. 3B6). Then the coatings are very difficult to be broken down, and the porosity goes down, the mean pore size stabilizes at a low level. That is why there is an inflection point in Fig. 4.

Furthermore, as the voltage is stable in the last stage of MAO processes, the discharge channels are stable accordingly. Nevertheless, the oxidation products formed per unit channel are increasing affected by the NCNs. Hence, the crateriform protuberances are formed finally, which results in the rise of surface roughness (Fig. 5). However, it is worth noting that there is an inflection point in surface roughness when the concentration of NCNs reach 1.2 g/L. As mentioned above, the NCNs have the effects of increasing the number of oxidation products formed per unit channel, and evenly distributing the surface current and micro-discharges. Therefore, when the NCNs reaches 1.2 g/L, the multifaceted role of NCNs achieves a balance point, and a compact MAO coating with lower surface roughness is formed.

### The composition of MAO coatings

**Figure 6:**
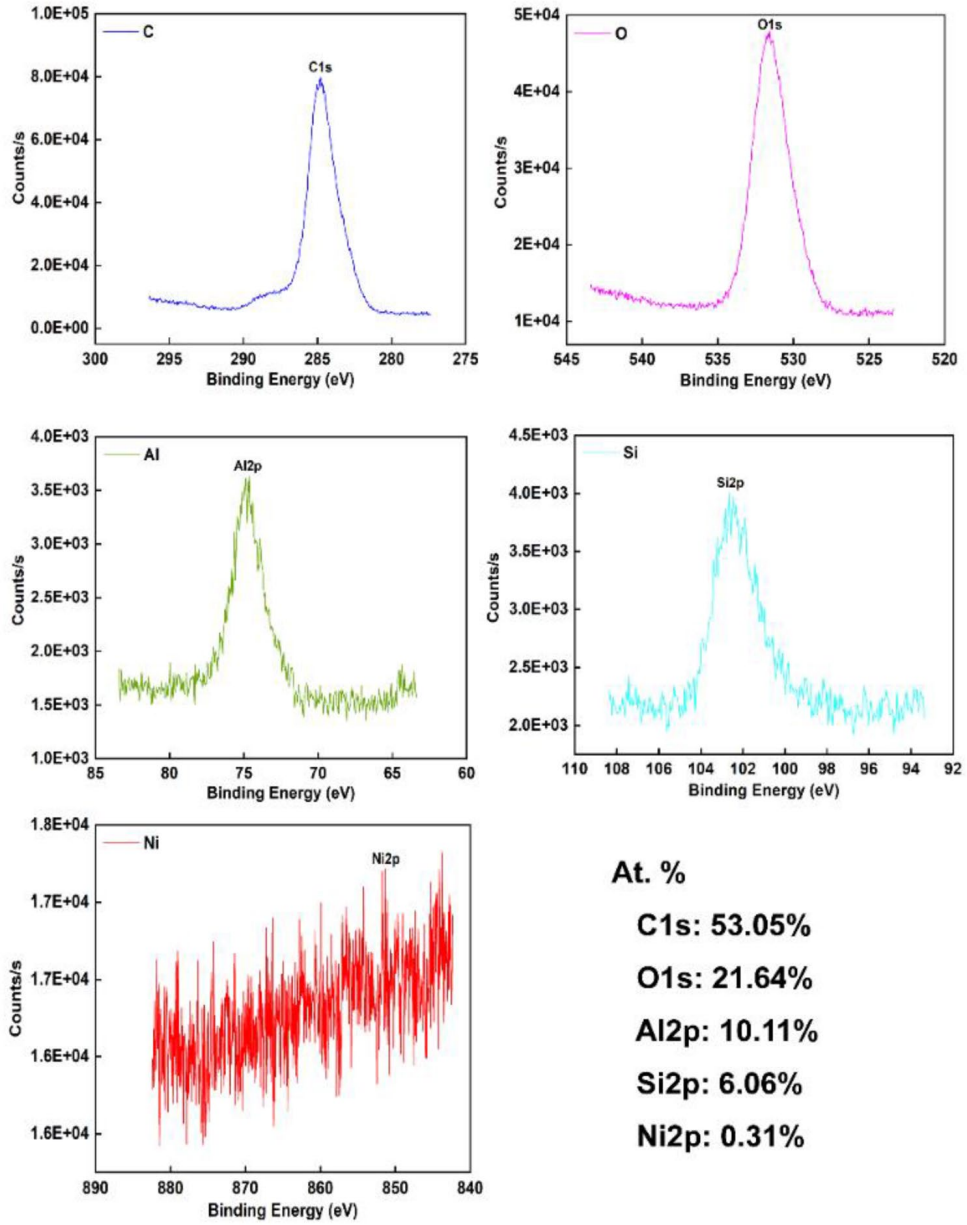
XPS results of the MAO coating prepared by 1.2 g/L NCNs (OriginPro 2018, https://www.originlab.com/index.aspx?go=Products/Origin; Adobe Illustrator CS5, https://www.adobe.com/cn/products/illustrator.html#scroll).

**Figure 7:**
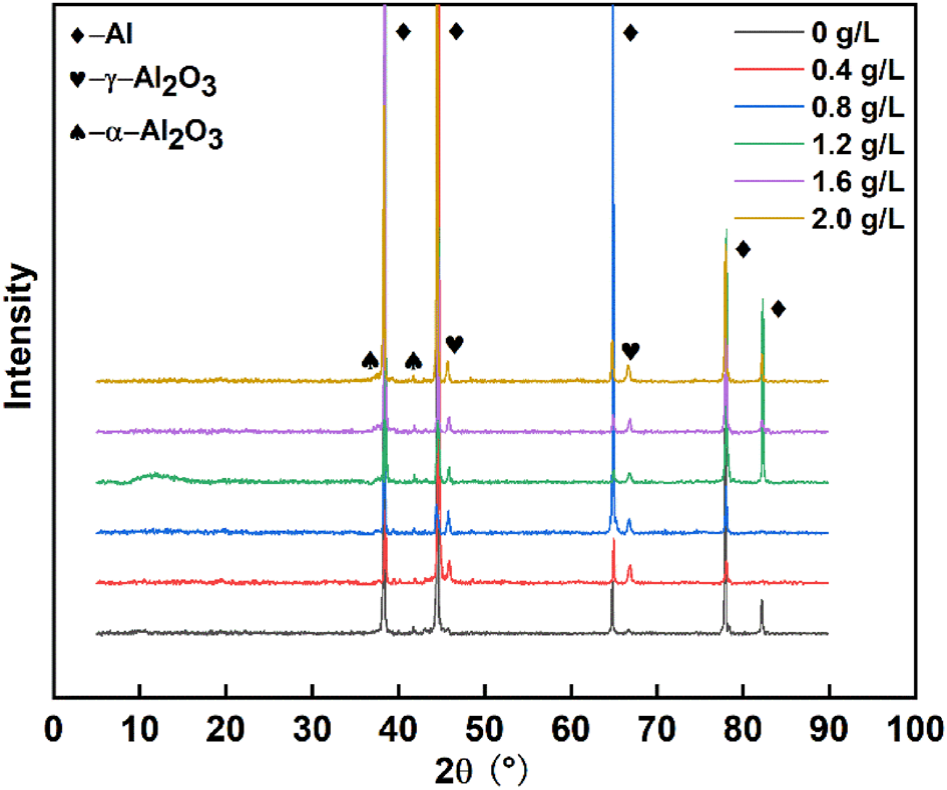
XRD results of the MAO coatings with different concentrations of NCNs (OriginPro 2018, https://www.originlab.com/index.aspx?go=Products/Origin).

The surface chemical element analysis result of the MAO coating prepared by 1.2 g/L NCNs is shown in Fig. 6. The phase composition analysis result of MAO coatings is shown in Fig. 7. As shown in Fig. 6, the content of Ni is extremely low. Therefore, although the NCNs possess a significant effect on the MAO reaction process, they are not visibly involved in the formation of MAO coatings. The surface is mainly composed of O, Al, Si. The O is from the electrolyte, the Al is from ZL109 substrate, and the Si is from electrolyte and substrate. The C mainly comes from external pollution.

**Figure 8:**
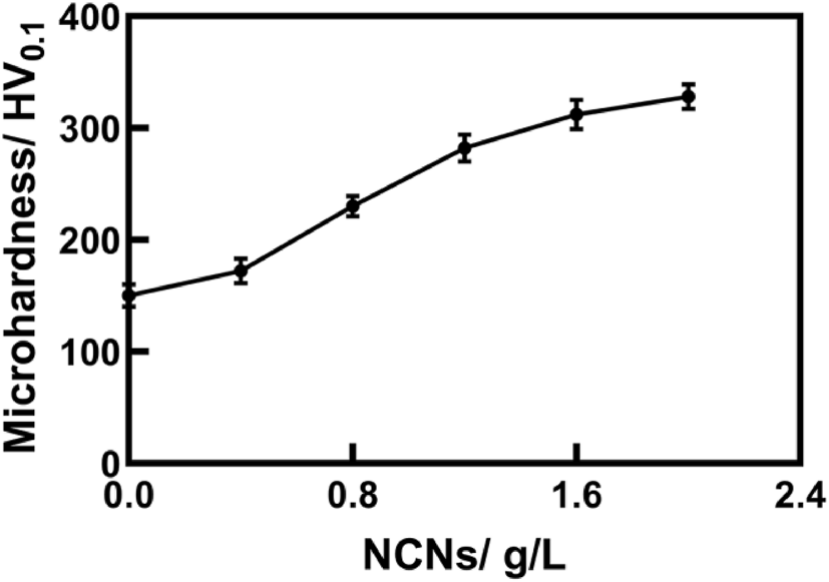
The surface microhardness of MAO coatings (OriginPro 2018, https://www.originlab.com/index.aspx?go=Products/Origin).

The phase composition and surface element analysis results form a mutual confirmation. The MAO coatings mainly contain α-Al_2_O_3_ and γ-Al_2_O_3_. As the coatings are relatively thin, and X-ray has strong penetrating power, there are some obvious diffraction peaks of Al in Fig. 7. Additionally, it can be found that the sample without NCNs has unconspicuous peaks of α-Al_2_O_3_ and γ-Al_2_O_3._ As shown in Fig. 5, the thickness of the coating without NCNs is relatively small, therefore, the intensity of α-Al_2_O_3_ and γ-Al_2_O_3_ is reduced accordingly. Furthermore, there is some small difference among the other results. For instance, the intensity of γ-Al_2_O_3_ of the coating with 1.2 g/L NCNs exhibits the lowest level, and that of α-Al_2_O_3_ exhibits a relatively higher level, which can be explained through the high storage dielectric micro-charging ability of NCNs. As mentioned above, while the concentration of NCNs reaches a certain level in the electrolyte, the MAO process gets a wonderful state, which is good for γ-Al_2_O_3_ transformed to α-Al_2_O_3_, and the MAO coating becomes more compact. Then, the microhardness of coatings is also increased accordingly, the details are shown in Fig. 8. When the concentration of NCNs is higher than 1.2 g/L, the coatings can not be dramatically broken down under the constant power supply voltage. Therefore, the trend of hardness increase slows down. However, as the energy of the reaction has reached a certain bottleneck, the amount of γ-Al_2_O_3_ goes up again with crateriform protuberances appearing.

### Friction-wear tests

**Figure 9:**
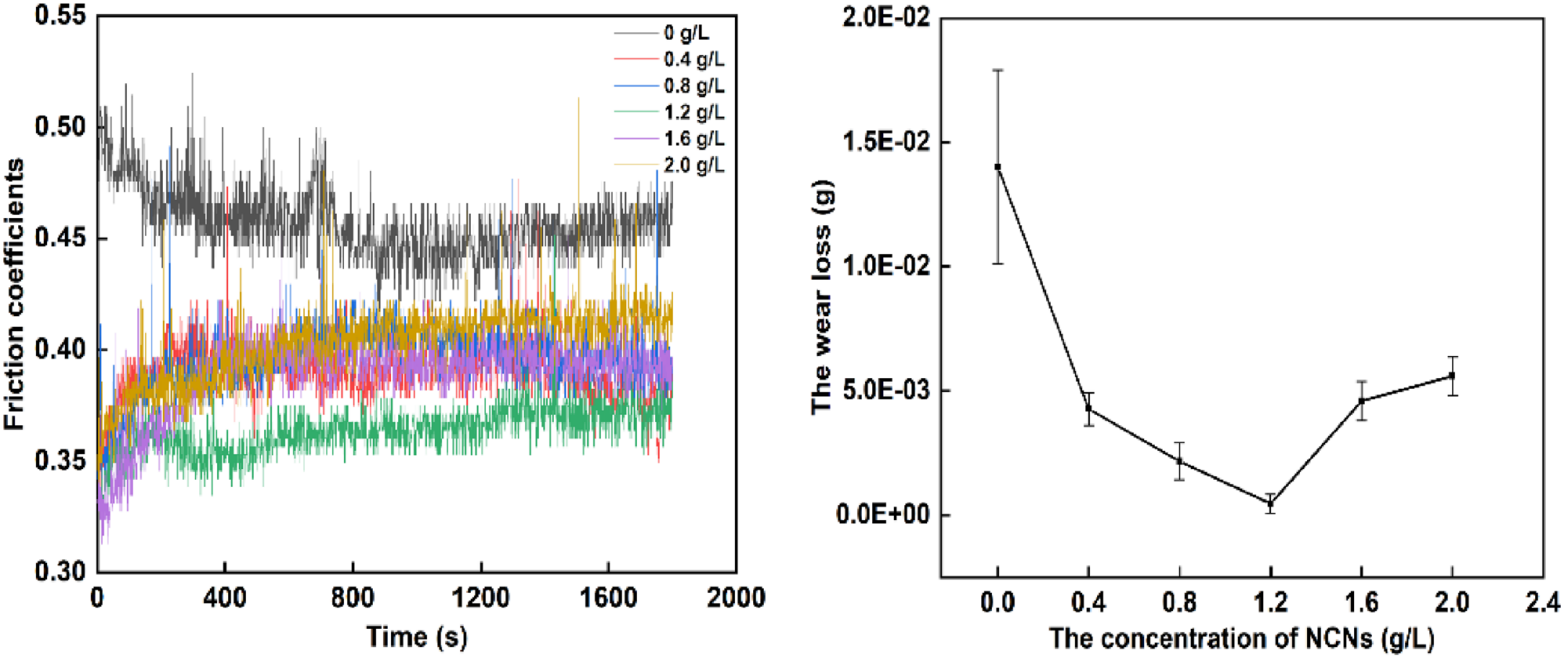
The friction coefficients and wear loss of MAO coatings (OriginPro 2018, https://www.originlab.com/index.aspx?go=Products/Origin; Adobe Illustrator CS5, https://www.adobe.com/cn/products/illustrator.html#scroll).

**Figure 10:**
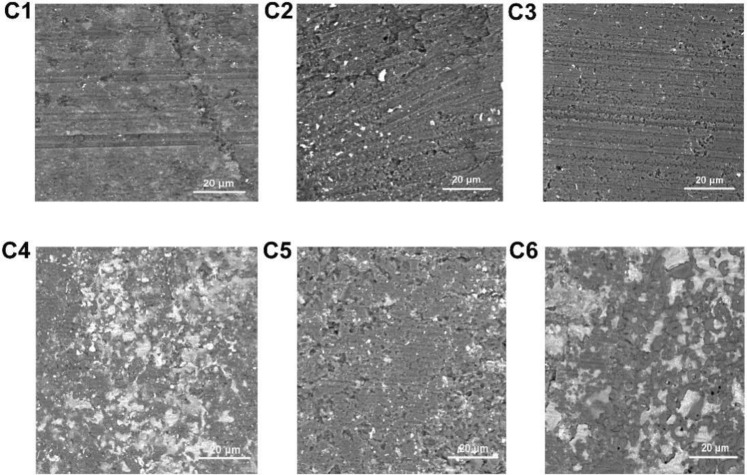
The worn surface morphology of MAO coatings (**C1**): The concentration of NCNs is 0 g/L; (**C2**): 0.4 g/L; (**C3**): 0.8 g/L; (**C4**): 1.2 g/L; (**C5**): 1.6 g/L; (**C6**): 2.0 g/L; Adobe Illustrator CS5, https://www.adobe.com/cn/products/illustrator.html#scroll).

Friction-wear tests are carried out to analyze the wear resistance of the MAO coatings affected by the NCNs. The wear loss of MAO coatings and friction coefficients are shown in Fig. 9. The worn surface morphology of MAO coatings is shown in Fig. 10.

As shown in Fig. 9, the MAO coating without NCNs exhibits the highest friction coefficient, the coating prepared by 1.2 g/L NCNs possesses the lowest friction coefficient, the others have little difference in friction coefficients. Although the coating without NCNs possesses lower surface roughness than the others, its thickness is thinner and more porous. Therefore, in the friction-wear tests, the coating without NCNs is easy to be destroyed by the load, and exhibits severe scratches (Fig. 10), high wear loss and friction coefficients (Fig. 9). Conversely, the coating prepared by 1.2 g/L NCNs is more compact, and thick with lower surface roughness (Fig. 5). Hence, the coating (1.2 g/L NCNs) exhibits more smother worn surface and lower friction coefficients. On account of the crateriform protuberances formed on the surface (Fig. 2), the wear loss increases gradually when the concentration of NCNs is higher than 1.2 g/L (Fig. 9). The results of sclerometric tests show that the binding force between the coating with 1.2 g/L NCNs and substrate reaches 50 N, which is higher than that of the coating without NCNs (30 N). Although the coatings with 1.6 g/L and 2.0 g/L NCNs have a little higher binding force (50–60 N), the crateriform protuberances formed are not suitable for wear resistance.

**Figure 11:**
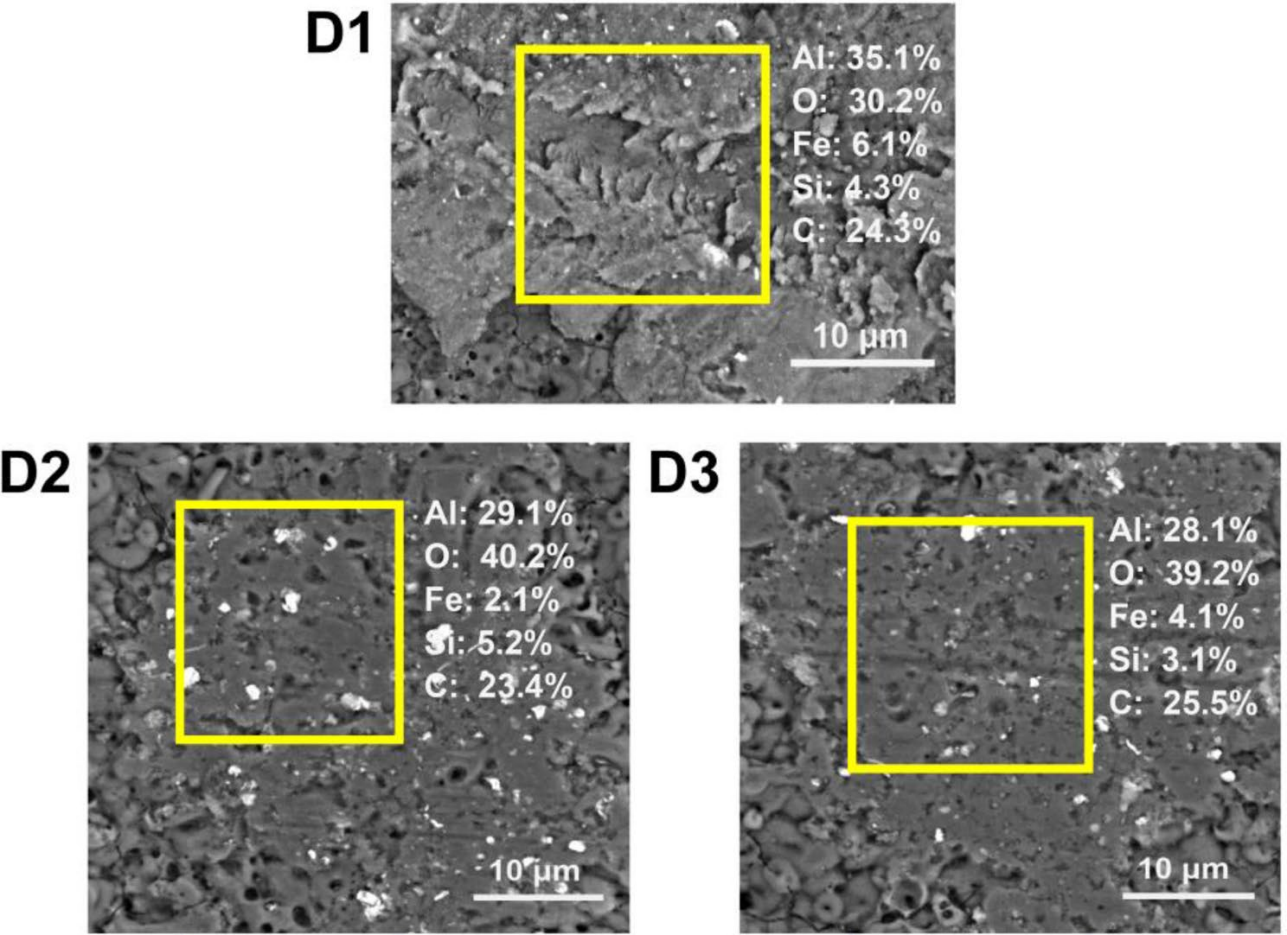
Partial worn surfaces and EDS results of the coatings (**D1**): the coating without NCNs, (**D2**): the coating with 1.2 g/L NCNs, (**D3**): the coating with 1.6 g/L NCNs; Adobe Illustrator CS5, https://www.adobe.com/cn/products/illustrator.html#scroll).

Furthermore, partial worn surface details with high magnification are shown in Fig. 11. As shown in Fig. 11D1, the surface of the coating without NCNs exhibits obvious scratches of abrasive wear. However, when the NCNs is introduced, the microhardness of coatings increases and the coatings becomes more compact, so the scratches are reduced dramatically, especially, the worn surface of the coating with 1.2 g/L NCNs is very flat. Additionally, there are some relatively slight scratches appearing again on the surface of the coating with 1.6 g/L NCNs, they are caused by the crateriform protuberances (introducing more hard particles during friction-wear process) (Fig. 2A5). That is why that the wear loss goes up when the concentration of NCNs is higher than 1.2 g/L.

The EDS results of the partial worn surfaces show that the worn surfaces mainly contain Al, O, Fe, Si, and C. Compared with the XPS results, Fe is from the sliding counterbody (boron copper cast iron). D1 has higher content of Al and Fe, which can be explained that the friction-wear process was very drastic, even partial coating was destroyed, the substrate (ZL109) exposed in partial area, and more Fe is transferred to the mating surface from the counterbody. When the scratches are reduced, the content of Fe goes down accordingly, therefore, the Fe is transferred from the counterbodies. To sum up, the coating prepared by 1.2 g/L NCNs possesses better wear resistance property than the others. Additionally, compared with the most excellent coating of literature^9^, the reduction of mean pore size reaches up to 52.5%, the reduction of porosity reaches up to 68%, the coating is more compact, and the wear loss has little difference. Therefore, the NCNs significantly improved MAO coatings.

## Conclusion

In this work, the effect of nickel-coated carbon nanotubes on the morphology, composition, and wear resistance of ZL109 aluminum alloy MAO coatings is analyzed systematically. The analysis results indicate that:

1. The NCNs have a significant influence on the pore size, porosity, thickness, and surface roughness of MAO coatings by affecting the number of oxidation products formed per unit channel, and distribution of surface current and micro-discharges.
2. The chemical composition of coatings affected by NCNs possesses similar characteristics with the coating without NCNs, and the NCNs are not directly involved in the formation of MAO coatings.
3. There is a critical concentration of NCNs for MAO coatings in the MAO electrolyte. Under this work, when the NCNs concentration is 1.2 g/L, the effect of NCNs reaches an excellent balance point. The coating prepared by 1.2 g/L exhibits outstanding integrative properties, including morphology and wear resistance.

## Data Availability

The datasets used and analyzed during the current study available from the corresponding author on reasonable request.
